# Fibrinous Pericarditis in the Horse, with Purpura Hæmorrhagica

**Published:** 1881-01

**Authors:** 


### V.—A CASE OF FIBRINOUS PERICARDITIS IN THE HORSE, WITH ENORMOUS EFFUSION, ASSOCIATED WITH PURPURA HEMORRHAGICA, AND FOLLOWING THE "EPIZOOTIC" CATARRH.

---

The following case was under the charge of Dr. F. V. Walton, veterinary surgeon, for the first few days prior to admission to the Columbia Veterinary College Hospital, where the case came directly under the care of N. F. Thompson, D. V. S.

The post-mortem was worked up by Drs. T. E. Satterthwaite and W. H. Porter, and the heart, with pericardial sac, both of which were covered with a most exuberant growth of sillosities, constituting the condition known as "hairy heart," and presented at the Pathological Society of New York. Gray mare, aged eighteen years, weighing about 1,200 lbs., was first seen October 11, 1880. She then had marked œdema of both hind extremities, and slight œdema of the fore ones. She had previously suffered from the "epizootic," though mildly, and it was thought she had recovered. The visible mucous membranes at this first inspection were very much congested, and marked by numerous blotches, indicating extravasations of blood. Temperature, 102.4° F.; pulse 44; respiration, 15. The animal was now eating well, and the bowels were regular. The symptoms, however, did not improve, and hemorrhagic spots were soon found on the inner sides of the thighs and under the skin in other parts. The diagnosis of *purpura hemorrhagica* had been made.

When next seen the temperature had risen to 104° F., and the pulse had fallen to 42; respiration 15. The general condition had not improved, and there was a serous oozing from some collections of œdema on the extremities.

On October 15th the mare was found to be failing. The œdema had mounted to the body, and was apparent on the nose, where the characteristic line of demarcation between the serous collections and uninvaded tissue was well seen and sharply defined, as is usual in purpura. The respiration was now 25, and much embarrassed, owing to the external swelling. Temperature was 106° F., pulse 50. The animal was greatly depressed and debilitated. On October 16th, the swelling of the head had become so great that it was almost impossible for the respirations to be carried on. Accordingly, tracheotomy was performed (10 A.M.) by Dr. N. F. Thompson, veterinary surgeon. The asphyxiated condition was at once relieved, and respiration became almost normal in frequency. The appetite also returned. Digitalis was now administered, to control the heart and remove the œdema.

On October 20th, this object appeared to have been accomplished quite satisfactorily, so on that day the tracheotomy tube was removed. The condition of the animal seemed to be fairly good, though there was great exudation of serum from the posterior extremities, and some ulceration and even sloughing of the skin. This latter unfortunate condition progressed so that the lower part of the extremities were simply covered with ulcerations. At this time the urine was examined, but though it contained the carbonate and phosphate of lime in abundance, together with some pus, there was no more albumen than would be accounted for by the latter.

The pulse now became very rapid and feeble (80), but the mare continued eating and walking about. Suddenly, at 11 P.M., she fell, and was found to have instantly died. A post-mortem examination was subsequently made by Messrs. Wallace and Frey, students of veterinary medicine.

On removing the sternum, the pericardium was found firmly attached to it and to the sternal end of the ribs on the right side, leading at first to the erroneous supposition that it was a case of pleuritic adhesion.

Upon cutting into the pericardial sac, it was found to contain an enormous quantity of slightly turbid serous fluid, ranging from nine to twelve gallons. This distended sac had displaced and compressed the right lung much more than the left. The whole inner surface of the pericardium was covered with a thick layer of villous projections on both visceral and parietal surfaces. To the naked eye the heart looked much like an enormous sponge that had been soaked in water. The layers of fibrinous material were neither very thick nor very firmly attached, which would imply that the disease had not existed a long time. The weight of heart, with its contained coagula and the pericardium, was nineteen pounds—an excess of ten pounds over the weight of a normal empty heart. Under the microscope, the villosities were found to be partially organized, and contained an abundance of crystals of a brown color, either in rhombic plates or in needles, which latter were often arranged in stellate fashion. The rhombs were regarded as hæmoglobine crystals, and the imperfect rosettes as hæmatoidine. The *lungs* were normal, except for œdema and hypostatic congestion. Beneath the mucous membrane of the *stomach*, as well as in other organs, there were numerous hemorrhagic spots. The *spleen* was soft; so also were the *liver* and *kidneys*. These latter were almost diffluent, and on microscopic examination exhibited very granular epithelia, indicating parenchymatous difficulty, such as is met with in all diseases where the blood is profoundly infected.

Dr. Satterthwaite observed that, *apropos* of the connection between epidemic diseases of the lower animals and man—a subject that was now attracting much attention—it was well to know that this disease was thought to be closely allied to another disease in the horse, known as *scarlatina*. Indeed, several eminent English veterinary surgeons had thought it impossible always to distinguish between the two. Others, again, had regarded it as a carbuncular disease of the anthrax or charbon family. Since Copland, according to Williams—one of the most recent systematic writers on veterinary medicine—had stated that scarlatina originated in the horse, and had been communicated from him to man and other animals, the disease was worth some attention. It could not be said, however, that there was any marked parallelism between scarlatina in the horse and man.

Still, if we accept the description given by Williams, it would be observed that the petechial eruption, which latter is confluent, corresponds to the human type; then, œdema is common to both, and so are sloughing of the skin, rheumatism, heart complications, and extravasations of blood. The œdema, however, is not thought to be renal, and the disease is intermittent, or, rather, remittent, in character, having curious exacerbations. It is also thought to be non-contagious.

This particular disease was *purpura*, and it exhibited the characteristic appearances that have been described. Fibrinous pericarditis was thought to have been excited by the general blood disease.

In answer to a question, Dr. Linntard stated that purpura hemorrhagica was a common sequillan of the epizootic, and occurred generally as an evidence of a broken-down condition in horses that were kept at work during the progress of the disease. The purpura was the direct cause of death in a large proportion of those cases.

